# Dyslipidemia is associated with sarcopenia of the elderly: a meta-analysis

**DOI:** 10.1186/s12877-024-04761-4

**Published:** 2024-02-23

**Authors:** Bingqing Bi, Xinying Dong, Meilin Yan, Zhuo Zhao, Ruitong Liu, Shugang Li, Hao Wu

**Affiliations:** 1 School of Public Health, Capital Medical University, Beijing, 100069 China; 2 School of Traditional Chinese Medicine, Capital Medical University, Beijing, 100069 China; 3 School of General Practice and Continuing Education, Capital Medical University, Beijing, 100069 China

**Keywords:** Sarcopenia, Dyslipidemia, TC, HDL-C, LDL-C, TG

## Abstract

**Purpose:**

Sarcopenia is a pathological change characterized by muscle loss in older people. According to the reports, there is controversy on the relationship between dyslipidemia and sarcopenia. Therefore, this meta-analysis aimed to explore the association between sarcopenia and dyslipidemia.

**Methods:**

We searched the Cochrane Library, Web of Science, PubMed, China National Knowledge Infrastructure (CNKI), Wan Fang, China Science and Technology Journal Database (VIP Database) for case‒control studies to extract data on the odds ratio (OR) between sarcopenia and dyslipidemia and the MD(mean difference) of TC, LDL-C, HDL-C, TG, and TG/HDL-C between sarcopenia and nonsarcopenia. The JBI(Joanna Briggs) guidelines were used to evaluate the quality. Excel 2021, Review Manager 5.3 and Stata 16.0 were used for the statistical analysis.

**Results:**

Twenty studies were included in the meta-analysis, 19 of which were evaluated as good quality. The overall OR of the relationship between sarcopenia and dyslipidemia was 1.47, and the MD values of TC, LDL-C, HDL-C, TG, and TG/HDL-C were 1.10, 1.95, 1.27, 30.13, and 0.16 respectively. In female, compared with the non-sarcopnia, the MD of TC, LDL-C, HDL-C, TG of sarcopenia were − 1.67,2.21,1.02,-3.18 respectively. In male, the MD of TC, LDL-C, HDL-C, TG between sarcopenia and non-sarcopenia were − 0.51, 1.41, 5.77, -0.67. The OR between sarcopenia and dyslipidemia of the non-China region was 4.38, and it was 0.9 in China. In the group(> 60), MD of TC between sarcopenia and non-sarcopenia was 2.63, while it was 1.54 in the group(20–60).

**Conclusion:**

Dyslipidemia was associated with sarcopenia in the elderly, which was affected by sex, region and age.

## Introduction

Sarcopenia is defined as the loss of muscle mass and strength which is a comprehensive, progressive pathological change characterized by a reduction in skeletal muscle and a decrease in physical activity in older people.There are differences in body composition and diagnostic methods among people in different regions. The Asia criteria of sarcopenia is (1) grip strength: Male < 28 kg, female < 18 kg (2) 6-meter walking test < 1.0 m/second (3) Simplified Physical Function Assessment (SPPB) ≤ 9 points (4) 5 sit up tests for ≥ 12 s (5) Dual energy X-ray method: male < 7.0 kg/m^2^, female < 5.4 kg/m^2^. The European criteria of sarcopenia was: (1) Grip strength: Male < 27 kg Female < 16 kg (2) 6-meter walking test < 0.8 m/second (3) Simplified Physical Function Assessment (SPPB) ≤ 8 points (4) 5 sit up tests for ≥ 15 s. (6) Muscle mass:<7.0 kg/m^2^ < 5.5 kg/m^2^. A study [1] from Europe reported that the prevalence of sarcopenia varied from 10% to 27% of people (≥ 60) worldwide, and the prevalence of severe sarcopenia ranged from 2% to 9%. In addition, the prevalence of sarcopenia in older people in Shanghai was 22.9%. Recently, some studies found that the prevalence of dyslipidemia in the elderly was 53.65%, and there was approximately 40% dyslipidemia globally [2]. It was inllustrated that higher TG can elevate the level of ROS, GDF-8, SIR1 and then decrease the synthesis of muscle and ROS is a risk factor of the cardiocascular disease [3].

Some studies have indicated that the association between sarcopenia and dyslipidemia is controversial. To date, data from a study [4] illustrated that the LDL-C of females with sarcopenia was 3.1 mg/dL more than that of non-sarcopenic individuals, and a study by Zhang et al. [5] found that dyslipidemia was associated with increased odds of sarcopenia(OR = 1.506, 95% CI: 1.112 ~ 2.140). However, a study [6] including 1543 older individuals indicated that compared with non-sarcopenic people, the levels of VLDL-C (0.49 ± 0.18 mmol/L), TG (1.57 ± 0.92 mmol/L), and TG/HDL-C (1.26 ± 1.10 mmol/L) in sarcopenic people were significantly lower (*P* < 0.05). Moreover, a study performed by Yushu Guo [7] demonstrated that there was no significant difference in HDL-C and LDL-C between sarcopenia and non-sarcopenia people. These studies showed a controversial relationship between dyslipidemia and sarcopenia, which prompted us to conduct a meta-analysis to explore the relationship. The results would shed more light on the prevention and treatment of sarcopenia and improving the health condition of older people.

## Methods

This research was conducted according to the Preferred Reporting Items for Meta-Analysis (PRISMA) guidelines.

### Literature search

Web of Science, PubMed, Cochrane Library, China National Knowledge Infrastructure (CNKI), Wan Fang, China Science and Technology Journal Database (VIP Database) were searched studies demonstrating the association between sarcopenia and dyslipidemia published through August 2023. Our search terms included “sarcopenia (MeSH)” OR “muscle (reduce OR loss)” AND “dyslipidemia (MeSH)” OR “(abnormal OR high) AND (blood fat OR lipid)” OR “TC” OR “LDL-C” OR “HDL-C” OR “TG” OR “TG/HDL-C.”

### Selection criteria

The inclusion criteria were as follows: (1) literature published in English or Chinese; (2) topic of studies relevant to the relationship between sarcopenia and dyslipidemia; (3) the variables of the studies including “TG”, “TC”, “HDL-C”, “LDL-C”; and “OR” of sarcopenia and dyslipidemia” (4) applicable data can be extracted.

Duplicate publications, reviews, case reports, literature not published in English or Chinese, literature with inconsistent experimental subjects, inappropriate methods or not including the variables this research needs or necessary data were excluded. Disagreements were resolved by discussion with all investigators.

### Data extraction and confirmation

First author, publication year, sample size, odds ratio of sarcopenia and dyslipidemia, the mean values of TC, LDL-C, HDL-C, TG, TG/HDL-C in sarcopenia and non-sarcopenia people, location, gender, age and the criteria of sarcopenia and dyslipidemia were collected in the eligible literature with a standardized diagram.

### Literature quality evaluation

Two reviewers (Bingqing Bi and Xinying Dong) evaluated the included literature to illustrate the validity of any findings investigated through the JBI(Joanna Briggs) [8] guidelines. The scoring criteria are as follows: ① 0 points: not meeting the requirements; ② 1 point: mentioned but not described in detail; ③ 2 points: detailed, comprehensive, and accurate description.When two reviewers have different opinions on the same article, we will discuss and propose solutions with Shugang Li.

### Statistical analysis

Excel 2021 was used for unit conversion of TC and LDL-C. HDL-C and TG from mol/L to mg/dl. Meta-analysis of the OR(Odds Ratio) between sarcopenia and dyslipidemia and the MD(Mean Difference) between TC, LDL-C, HDL-C, TG, TG/HDL-C and sarcopenia were performed by Review Manager 5.3. When I² was higher than 50%, the random effect model was used; when I² was less than 50%, the fixed effect model was performed. Subgroup analysis was carried out to explore the cause of heterogeneity. In addition, a funnel plot of the OR was conducted to assess whether there was publication bias in the research results, and a sensitivity analysis with Stata 16.0 was performed to evaluate the reliability of the results.

## Results

### Study selection

**Fig. 1:**
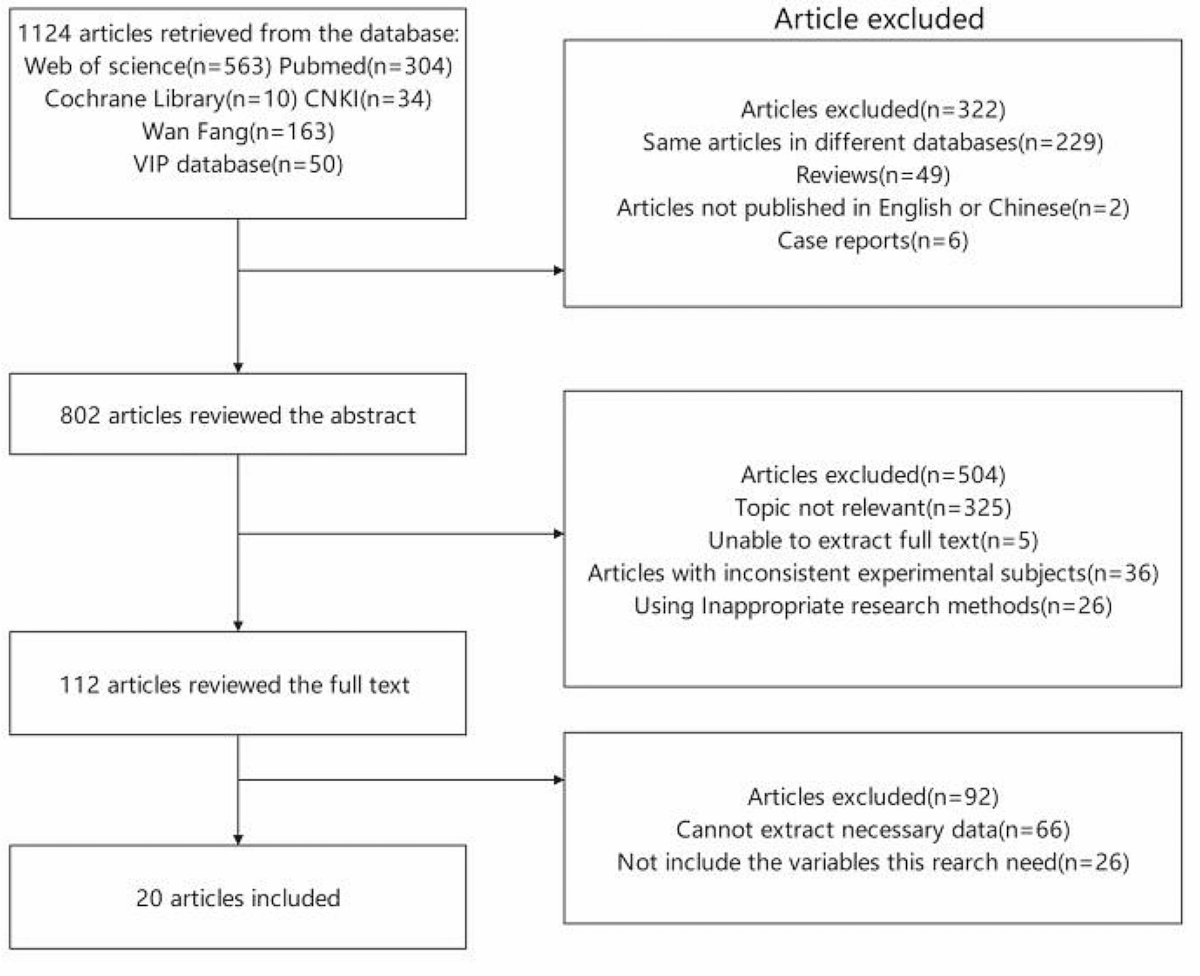
Flow chart of the selection process

The search process identified 1124 articles from 6 databases. Among them, 529 same articles in different databases, 49 reviews, 2 studies not published in English or Chinese, and 6 case reports were excluded by skimming the title. After reading the abstracts, the main reasons for exclusion were that the topic of the paper was not relevant to the association between sarcopenia and dyslipidemia, research was unable to extract full text, studies with inconsistent experimental subjects and inappropriate methods were used. Moreover, studies in which the necessary data could not be extracted and the variables this research needed were not included were excluded. Ultimately, twenty original case‒control studies [5, 6, 7, 9–25] met the inclusion criteria. The article selection process is shown in Fig. 1.

### Study characteristics

**Fig. 2:**
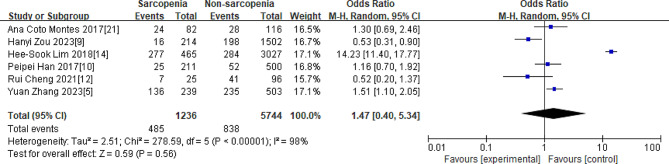
Overall OR of the relationship between sarcopenia and dyslipidemia

**Fig. 3:**
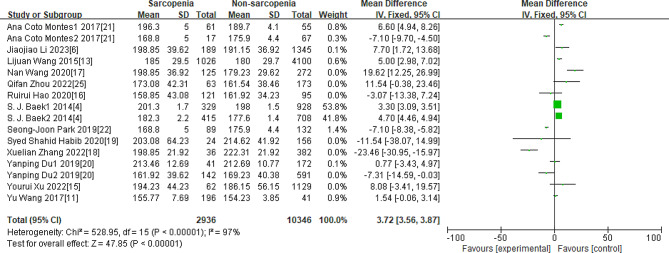
Overall MD of the average TC value between sarcopenia and nonsarcopenia people

**Fig. 4:**
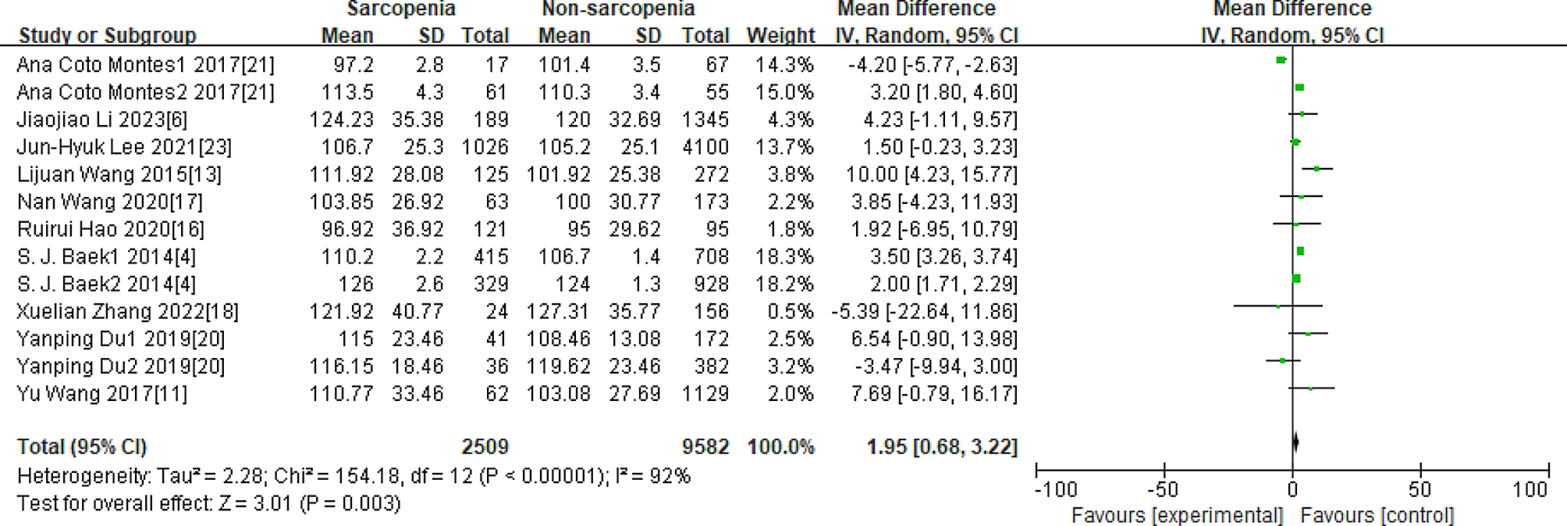
Overall MD of the average LDL-C value between sarcopenia and nonsarcopenia people

**Fig. 5:**
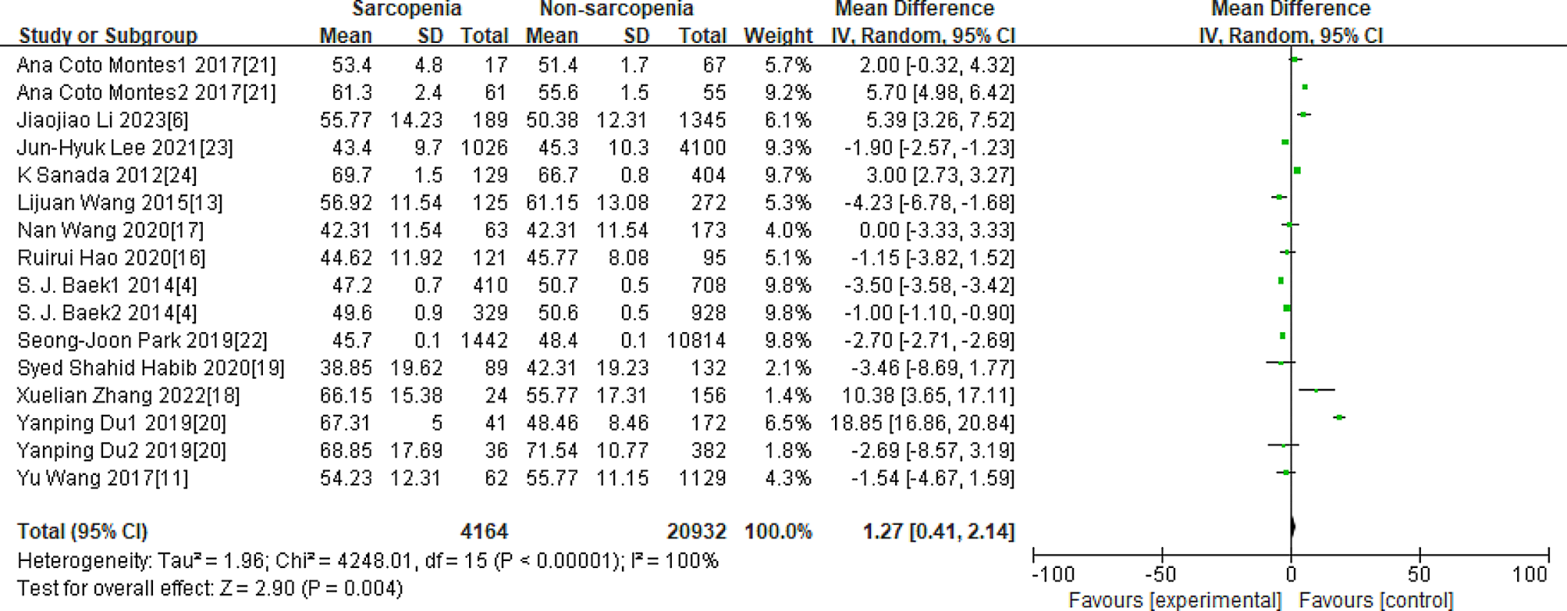
Overall MD of the average HDL-C value between sarcopenia and nonsarcopenia people

**Fig. 6:**
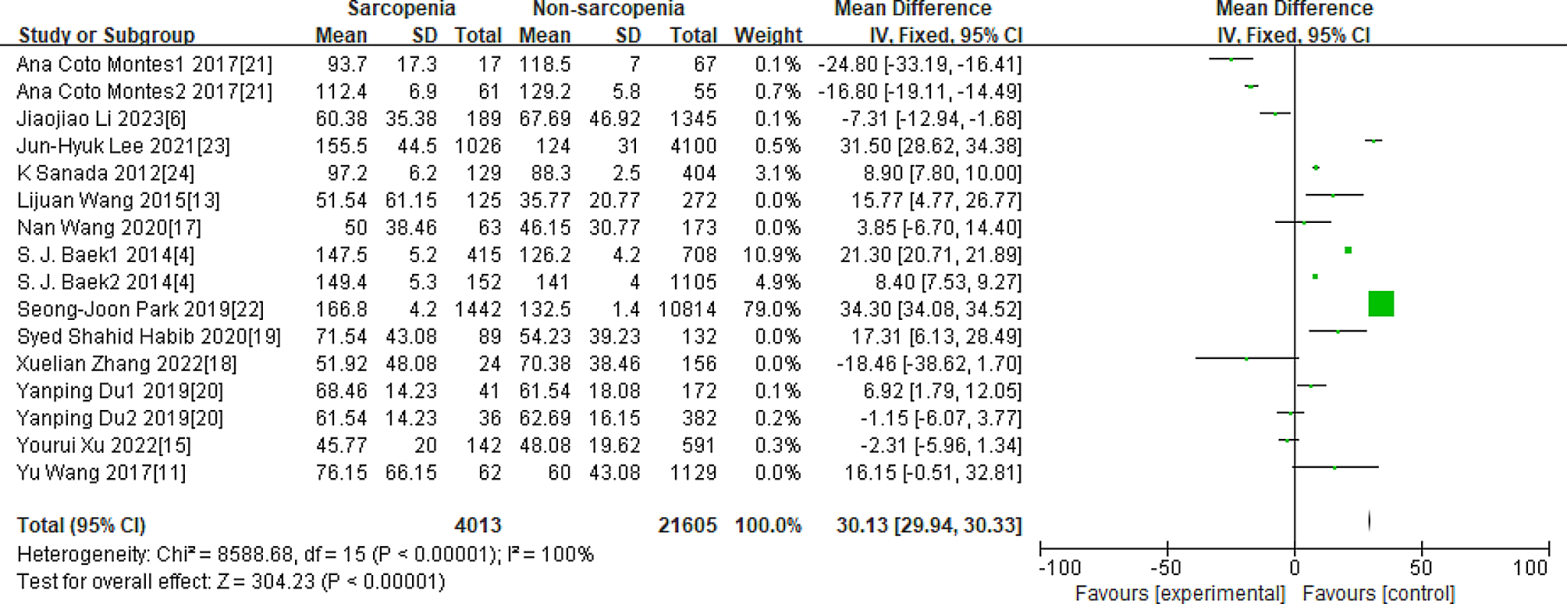
Overall MD of the average TG value between sarcopenia and nonsarcopenia people

**Fig. 7:**
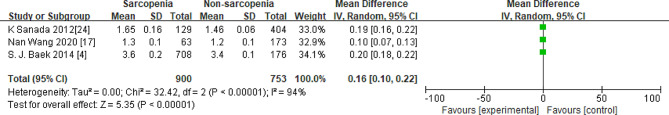
Overall MD of the average TG/HDL-C value between sarcopenia and nonsarcopenia people

The characteristics of studies are presented in Table 1, including the first author, country, age and the indicators this research includes. The studies were performed in America, China, Korea and Syrian Arab Republic, and only one subject was under 60 years old. However, the odds ratio of sarcopenia and dyslipidemia, the average values of TC, TG, HDL-C, LDL-C, and TG/HDL-C in sarcopenia and nonsarcopenia people and the sample size were manifested in the forest plot (Figures 2, 3, 4, 5, 6 and 7; Table 3).

**Table 1:** Characteristics of studies

No.	Research	Country	Age/years	The indicators this research include	Diagnostic criteria of sarcopenia	Diagnostic criteria of dyslipidemia
1	S. J. Baek [4]	Korea	≧ 65	2,3,4,5,6,7,9	Asia criteria1^a^	Criteria5
2	Yuan Zhang [5]	China	≧ 60	1,8	Asia criteria1^a^	Critertia1
3	Jiaojiao Li [6]	China	≧ 60	2,3,4,5,9	Asia criteria1^a^	Criteria3
4	Hanyi Zou [9]	China	≧ 60	1,8	Asia criteria1^a^	Criteria2
5	Peipei Han [10]	China	≧ 60	1,8	Asia criteria1^a^	Criteria4
6	Yu Wang [11]	China	≧ 60	2,3,4,5,9	Asia criteria2^d^	Criteria3
7	Rui Cheng [12]	China	≧ 60	1,8	Asia criteria1^a^	Criteria4
8	Lijuan Wang [13]	China	≧ 60	2,3,4,5	Asia criteria1^a^	Criteria4
9	Hee-Sook Lim [14]	America	≧ 60	1,8	Europena criteria^b^	Criteria6
10	Yourui Xu [15]	China	≧ 60	2,5	Asia criteria1^a^	Criteria2
11	Ruirui Hao [16]	China	≧ 60	2,3,4,9	Asia criteria3^e^	Criteria3
12	Nan Wang [17]	China	≧ 60	2,3,4,5,6,9	Asia criteria1^a^	Criteria2
13	Xuelian Zhang [18]	China	≧ 60	2,3,4,5	Asia criteria1^a^	Criteria3
14	Syed Shahid Habib [19]	Syrian Arab Republic	≧ 60	2,4,5	Syrian Arab Republic’s criteria^c^	Criteria6
15	Yanping Du [20]	China	≧ 65	2,3,4,5,7,9	Asia criteria1^a^	Criteria1
16	Ana Coto Montes [21]	Korea	≧ 60	1,2,3,4,5,7,8,9,	Asia criteria1^a^	Criteria6
17	Seong-Joon Park [22]	Korea	≧ 60	4,5	Asia criteria1^a^	Criteria7
18	Jun-Hyuk Lee [23]	Korea	≧ 60	2,3,4,5	Asia criteria1^a^	Criteria8
19	K Sanada [24]	Japan	≧ 60	4,5,6	Asia criteria1^a^	Ctiteria6
20	Qifan Zhou [25]	China	20–60	2,9	Asia criteria1^a^	Criteria2

Indicators:1:Overall OR,2:Overall TC MD,3:Overall LDL-C MD,4:Overall HDL-C MD,5:Overall TG MD,6:Overall TG/HDL-C, 7:Subgroup analysis of gender, 8:Subgroup analysis of area, 9:Subgroup analysis of age

^a^(i) skeletal muscle mass index (BIA) of the limbs: <7.0 kg/m2 for men < 5.7 kg/m2 for women; (ii) assessment of physical function: 6-meter stride < 1.0 m/s or SPPB score ≤ 9 or standing test ≥ 12 s; (iii) grip strength: <28 kg for men and < 18 kg for women; if ① and ② or③ were satisfied, the diagnosis of sarcopenia was made;

^b^Muscle mass decreased by dual energy X-ray absorptiometry (DXA)was divided by body weight and calculated as a percentage. When the calculated value was less than 1 standard deviation (SD), it was classified as sarcopenia.

^c^Authors analyzed appendicular lean mass (ALM) and hand grip strength test (HGS). Total and percent lean mass and TLM/ ht2 were calculated. ALM is the sum of arm and leg lean mass, and ALM/ht2 was also calculated. The sarcopenia was defined when the ALM/ht2 was less than 7.46.

^d^Skeletal muscle mass index (SMI) = Skeletal muscle mass/body weight ×100%, Sarcopenia is defined as a condition where the standard deviation is 2 times lower than that of the young control group (18–39 years old) of the same sex

^e^The calculation of skeletal muscle index (SMI) based on imaging to determine the presence of sarcopenia is calculated by dividing the total muscle area by the square of height. The threshold for diagnosing sarcopenia is less than 42.6 cm2/m2 for males and less than 30.6 cm2/m2 for females

Critertia1:TC ≥ 6.2 mmol/L、HDL-C ≥ 4.1 mmol/L、HDL-C ≤ 1.0 mmol/L、TG ≥ 2.3 mmol/L or use lipid-lowering drugs

Criteria2: TC ≥ 6.2 mmol/L、HDL-C ≥ 4.1 mmol/L、HDL-C ≤ 1.0 mmol/L、TG ≥ 2.3 mmol/L

Criteria3:LDL-C ≥ 3.37mmol/L, HDL-C<1.04mmol/L, TC ≥ 5.18mmol/L,TG ≥ 1.7mmol/L

Criteria4:TG ≥ 2.26 mmol/L, TC ≥ 6.22mmol/L, LDL -C ≥ 4.14 mmol/L, HDL-C ≤ 1.04mmol/L

Criteria5:TG > 400 mg/dL, HDL-C ≤ 160 mg/dL, TG/HDL-C>4

Criteria6: TG > 150 mg/dL, HDL-C < 40 mg/dL

Criteria7:TG > 150 mg/dL,HDL-C < 40 mg/dL for males or < 50 mg/dL for females or use of dyslipidemia medication

Criteria8: HDL-C < 40 mg/dL

### Methodological quality assessment

Joanna Briggs was conducted to evaluate the quality of the studies we included, and the results of the assessment are listed in Table 2. The fact that the score of 19 studies was more than 15 indicated that the quality of the studies we included was relatively better. The score of the study by Qifan Zhou were ≤ 1 in the aspect, including study population, the inclusion and exclusion criteria, sample features etc(② ③ ④ ⑥ ⑦ ⑧ ⑩, Table 2).

**Table 2:** Results of methodological quality assessment using the JBI

Number	Research	①	②	③	④	⑤	⑥	⑦	⑧	⑨	⑩	Overall
1	Ana Coto Montes	2	2	2	1	2	2	1	2	2	1	17
2	Hanyi Zou	2	1	2	1	1	1	1	2	2	1	14
3	Hee-Sook Lim	2	2	2	2	2	2	1	2	1	1	17
4	Jiaojiao Li	1	2	2	1	2	2	1	2	2	2	17
5	Jun-Hyuk Lee	2	2	2	2	2	2	1	2	2	1	18
6	K Sanada	2	2	2	1	1	2	1	1	2	2	16
7	Lijuan Wang	2	1	2	1	2	2	1	1	1	2	15
8	Nan Wang	1	2	2	1	2	2	1	2	1	1	15
9	Peipei Han	2	2	2	2	1	2	1	2	2	2	18
10	Qifan Zhou	2	1	1	1	2	0	1	1	2	1	12
11	Rui Cheng	2	2	2	2	2	2	1	2	2	2	19
12	Ruirui Hao	2	2	2	1	1	1	1	1	2	2	15
13	S. J. Baek	2	2	2	2	2	1	1	2	2	2	18
14	Seong-Joon Park	2	2	2	2	2	2	1	2	2	2	19
15	Syed Shahid Habib	2	2	2	2	2	1	1	2	1	2	17
16	Xuelian Zhang	2	1	2	2	2	1	1	2	1	1	15
17	Yanping Du	1	2	2	2	2	1	1	2	1	1	15
18	Yourui Xu	2	2	2	2	2	1	1	2	1	2	17
19	Yu Wang	2	2	2	2	2	1	1	2	1	2	17
20	Yuan Zhang	2	2	2	2	1	2	1	1	2	2	17

Evaluation criteria:① Is the research purpose clear? Is the basis for setting the question sufficient?②How was the study population selected (whether the study subjects were randomly selected, and whether stratified sampling was adopted to improve sample representativeness)?③Does clearly describe the inclusion and exclusion criteria for the sample?④Does clearly describe the sample features?⑤Does the tool for collecting data have reliability and validity (If using an investigator survey, how is the repeatability of the survey results)?⑥What are the measures to verify the authenticity of the information?⑦Does consider ethical issues?⑧Is the statistical method correct?⑨Are the statements of the research results appropriate and accurate? Are the results distinguished from the inference, and are the results faithful to the data rather than the inference?⑩Have you provided a clear explanation of the research value?

0 points: Not meeting the requirements; 1 point: mentioned but not described in detail; 2 points: Detailed, comprehensive, and accurate description

### Association between Sarcopenia and dyslipidemia

#### Overall OR of the relationship between Sarcopenia and dyslipidemia

As shown in Fig. 2, the overall OR of the relationship between sarcopenia and dyslipidemia was 1.47[0.40,5.34], Z = 0.59, *P* = 0.56.

#### Overall MD of TC, LDL-C, HDL-C, TG, and TG/HDL-C values between the Sarcopenia and non-sarcopenia

As Figs. 3, 4, 5, 6 and 7 were shown, the overall Mean Difference of the average TC, LDL-C, HDL-C, TG, TG/HDL-C value was 3.72[3.56,3.87](*P* < 0. 00001), 1.95[0.68,3.22](*P* = 0.003), 1.27[0.41,2.14](*P* = 0.004), 30.13[29.93,30.33](*P* < 0. 00001), 0.16[0.10,0.22](*P* < 0. 00001). The heterogeneity of the TC, LDL-C, HDL-C, TG, TG/HDL-C were significantly. Therefore, subgroup analysis was performed to analyze the origin of the heterogeneity.

Note for Figs. 3–7: Some studies only had the average values of TC, LDL-C, HDL-C, and TG in females and males, so we also included them in our research. The number “1” represents females, and the number “2” represents males. The unit of “mmol/L” was converted to “mg/dL”.

#### Subgroup analysis of gender, region and age

Subgroup analysis of sex, region and age is shown in Table 3. In female, compared with the non-sarcopnia, the MD of TC, LDL-C, HDL-C, TG of sarcopenia were − 1.67,2.21,1.02,-3.18 respectively. In male, the MD of TC, LDL-C, HDL-C, TG between sarcopenia and non-sarcopenia were − 0.51, 1.41, 5.77, -0.67. The OR between sarcopenia and dyslipidemia of the non-China region was 4.38, and it was 0.9 in China. In the group(> 60), MD of TC between sarcopenia and non-sarcopenia was 2.63, while it was 1.54 in the group(20–60).

**Table 3:** Subgroup analysis by gender, region and age

Factor	Indicator of the meta- analysis	Number of the research	Effect Variable	The result of the meta-analysis							
				95% CI	I²(%)	*Z*	*P*	95% CI	I²(%)	*Z*	*P*
				Female				Male			
Gender	TC	3	MD	-1.67 [-7.36,4.01]	97%	0.58	< 0.00001	-0.51 [-8.76,7.75]	98%	0.12	< 0.00001
	LDL-C	3	MD	2.21 [0.78,3.63]	63%	3.04	< 0.00001	1.41 [-4.98,7.79]	97%	1.69	< 0.00001
	HDL-C	3	MD	1.02 [-4.57,6.61]	99%	0.36	< 0.00001	5.77 [-8.13,19.66]	100%	3.47	< 0.00001
	TG	3	MD	-3.18 [-21.63, 15.28]	100%	0.34	< 0.00001	-0.67 [-11.49,10.14]	100%	0.12	< 0.00001
				China				Non-China			
Region	Total rate of the dyslipidemia	6	OR	0.90 [0.52,1.56]	98%	0.59	<0.00001	4.38 [0.41,5.34]	78%	0.38	0.004
				More than 60 years				Less than 60 years			
Age	TC	11	MD	2.63 [0.92,4.35]	98%	6.13	< 0.00001	1.54 [-0.89, 4.12]	-----	1.25	0.0002

#### Publication bias

**Fig. 8:**
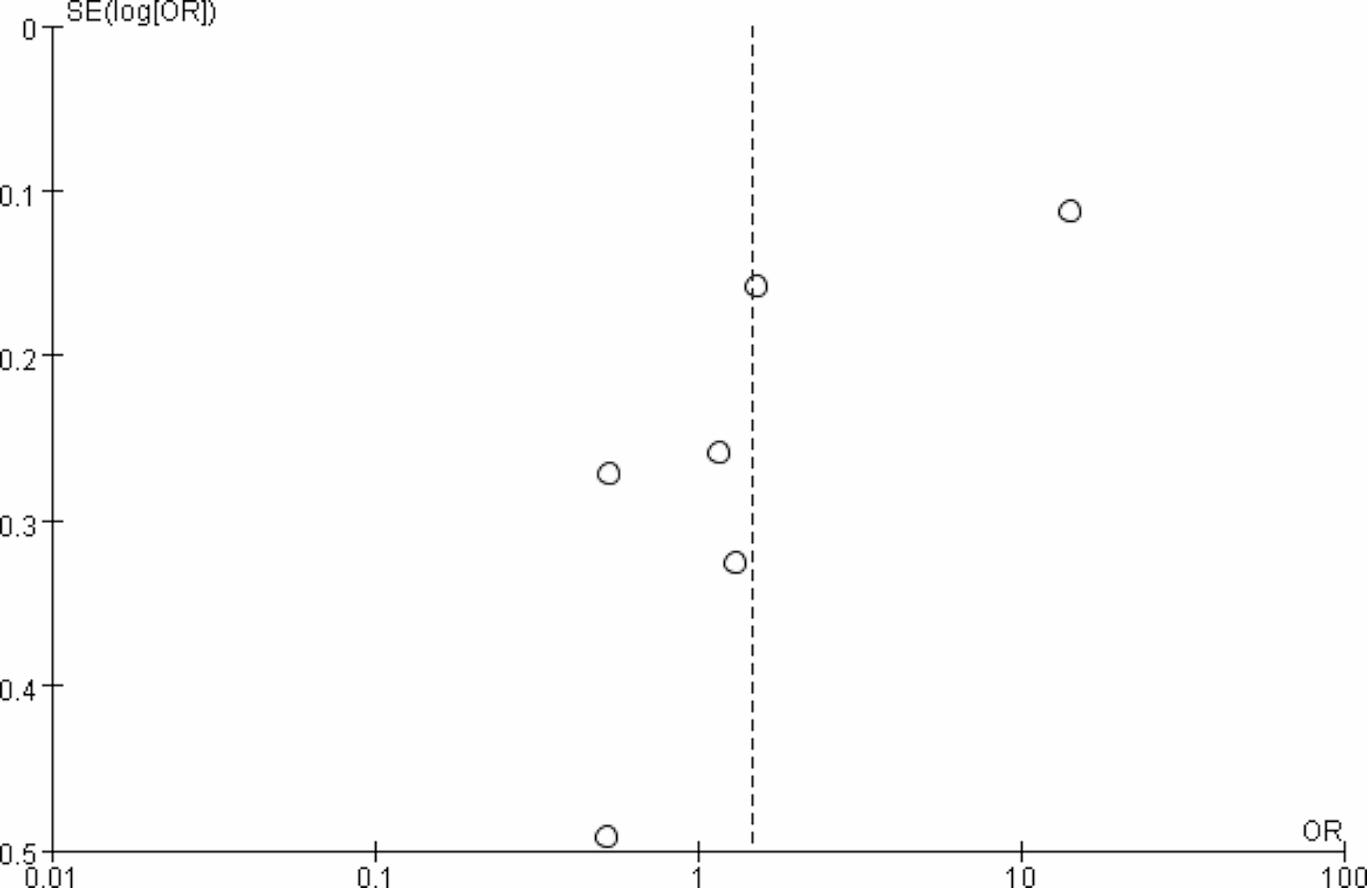
Bias analysis

The publication bias was presented in Fig. 8, which showed the asymmetry distribution of the articles. No significant bias was observed in this research.

#### Sensitivity analysis

**Fig. 9:**
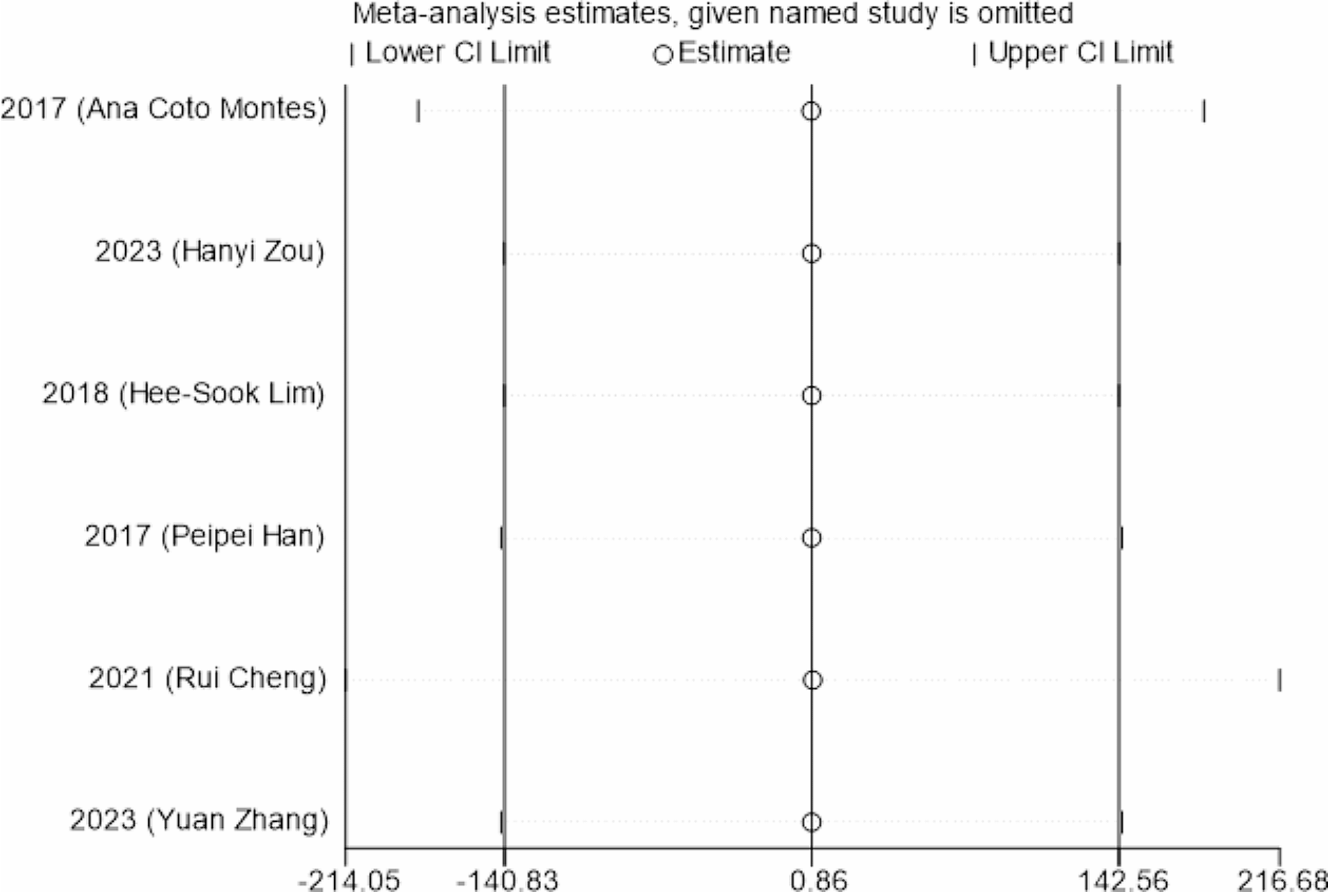
Sensitivity analysis

From Fig. 9, it was shown that the value of the sensitivity analysis was relatively concentrated, which demonstrated the reliability of the results.

## Discussion

The results of the present study showed that sarcopenia was positively associated with dyslipidemia. The overall OR was 1.47[0.40,5.34], and the total mean differences in TC, LDL-C, HDL-C, TG, and TG/HDL-C were 1.10[-0.50,2.71], 1.95[0.68,3.22], 1.27[0.41,2.14], 30.13[29.93,30.33], and 0.16[0.10,0.22] respectively, indicating that there was a difference in lipids between sarcopenia and non-sarcopenia patients.These results provided references for the prevention and control of sarcopenia.

In female, compared with the non-sarcopnia, the MD of TC, LDL-C, HDL-C, TG of sarcopenia were − 1.67,2.21,1.02,-3.18 respectively. In male, the MD of TC, LDL-C, HDL-C, TG between sarcopenia and non-sarcopenia were − 0.51, 1.41, 5.77, -0.67. It seemed that the difference between genders could be interpreted for many reasons, especially different hormone level [26]. With aging, the decrease in androgen levels led to less synthesis of skeletal muscle proteins. Correspondingly, the decreasing estrogen level may be associated with the rise of TNF-α, IL-6 and other inflammatory factors and then reduce the mass of the muscle [27]and the decrease of estrogen levels leads to mitochondrial dysfunction and muscle loss, mitochondrial division in satellite cells [28], the loss of energy can cause the electron transport chain (ETC) out of control, leading to low oxidative phosphorylation metabolism and mitochondrial phagocytosis efficiency, and increase oxidative stress, then decline the synthesis of muscle [29]. A study by Xiaoling Luo [30] stated that the decrease in estrogen levels affects the activity of multi-chain lipase and reduces the synthesis of HDL-C. Another study indicated that after menopause, women’s estrogen levels decreased and their inhibitory effect on liver enzymes weakened, leading to an increase in TG levels [31]. In addition, testosterone can affect the activity of tricarboxylic acid cycle enzymes, promote free fatty acids to enter the tricarboxylic acid cycle for oxidation, and reduce cholesterol synthesis [32]. Therefore, it could be inferred that the difference between androgen and estrogen affected the MD values of LDL-C, TG, HDL-C, and TG in females and males.

A study [33] indicated that the increase in lipids was proportional to the mass intake of red meat, fast food, such as clips [34], and sugary drinks, which is a habit of regions such as Europe and Korea [35]. The OR between sarcopenia and dyslipidemia of the non-China region was 4.38, and it was 0.9 in China. Compared with the non-China region, the Chinese relatively preferred the food made from wheat, flour, fruits and vegetables, which was in reverse ratio of increased lipid and European people ate the red meat more.

It was reported that C-reactive protein (CRP) was associated with degenerative changes, so CRP may be a driving factor of sarcopenia [36]. In the group(> 60), MD of TC between sarcopenia and non-sarcopenia was 2.63, while it was 1.54 in the group(20–60). In addition, compared with that of non-sarcopenic elderly people, the leptin level of sarcopenic elderly people was evidently increased [37].

It must be acknowledged that limitations existed in this meta-analysis.First, the diagnostic criteria of sarcopenia was different between Asia and Europe, which may be the origin of the heterogeneity. Second, the subgroup analysis of small metabolic molecules, such as P3NP, IL-6, TNF-α, and FGF-21, was not conducted as a result of the limited original studies. Moreover, all the included studies were case‒control studies, and the causality relationship could not be inferred.

## Conclusion

Dyslipidemia was associated with sarcopenia in the elderly, and sex, region and age were the influencing factors. Subsequent studies were proposed to elucidate the relationship between sarcopenia and dyslipidemia to explore the mechanism of the relationship between them.

## Data Availability

The datasets used during the present study are available from the corresponding author on reasonable request.
